# Background-based Delineation of Internal Tumor Volume in Static Positron Emission Tomography in a Phantom Study

**DOI:** 10.7508/aojnmb.2016.04.006

**Published:** 2016

**Authors:** Yangchun Chen, Xiangrong Chen, Ji-an Liu, Fanyong Li

**Affiliations:** 1 Department of Nuclear Medicine, Quanzhou First Hospital, Fujian Medical University, Quanzhou, China; 2 The PET-CT Center, First Affiliated Hospital of Guangzhou Medical University, Guangzhou, China; 3 Department of Radiology, Quanzhou First Hospital, Fujian Medical University, Quanzhou, China; 4 Guangdong Provincial Key Laboratory of Micro-nano Manufacturing Technology and Equipment, Guangdong University of Technology, Guangzhou, China

**Keywords:** Gross Tumor Volume, Internal Tumor Volume, Positron Emission Tomography, Standardized Uptake Value

## Abstract

**Objective(s):**

Considering the fact that the standardized uptake value (SUV) of a normal lung tissue is expressed as *x*±*SD*, *x*+3×*SD* could be considered as the threshold value to outline the internal tumor volume (ITV) of a lung neoplasm.

**Methods:**

Three hollow models were filled with 55.0 kBq/mL fluorine-18 fluorodeoxyglucose (^18^F-FDG) to represent tumors. The models were fixed to a barrel filled with 5.9 kBq/mL ^18^F-FDG to characterize normal lung tissues as a phantom. The PET/CT images of the phantom were acquired at rest. Then, the barrel was moved periodically to simulate breathing while acquiring PET/CT data. Volume recovery coefficient (VRC) was applied to evaluate the accuracy of ITVs. For statistical analysis, paired t-test and analysis of variance were applied.

**Results:**

The VRCs ranged from 0.74 to 0.98 and significantly varied among gross tumor volumes for delineating ITV (*P*<0.01). In two-dimensional PET scans, the motion distance did not affect VRC (*P*>0.05), whereas VRC decreased with increasing distance in three-dimensional PET scans (*P*<0.05).

**Conclusion:**

The threshold value (*x*+3×*SD*) had the potential to delineate the ITV of cancerous tissues, surrounded by lung tissues, particularly in two-dimensional PET images.

## Introduction

Based on the guidelines by the National Comprehensive Cancer Network (NCCN) on Non-Small Cell Lung Cancer (version 2, 2015), fluorine-18 fluorodeoxyglucose (^18^F-FDG) positron emission tomography (PET) has been recommended for delineating lung tumor target volume (1).

Since respiratory motions affect quantification in un-gated PET images (2), several studies have attempted to outline the gross tumor volume (GTV) and/or internal tumor volume (ITV) on PET images, acquired by gated or list-mode scans (3-6).

Several methods have been proposed for outlining lung neoplasms on PET images. These methods are based on the standardized uptake value (SUV) of the tumor alone or the SUV of the tumor combined with that of the background or even the tumor volume and motion distance (7).

Since the coincidence time window is less than 12 ns during PET scan (8), any coincidence event can be regarded as a free motion event. In other words, it can be stated that the coincidence event is immobile, while the lung tumor is in motion. Therefore, it was speculated that PET images of lung tumors at rest could be linearly translated and overlaid to simulate un-gated PET images during breathing. Un-gated PET images of a lung tumor should accurately reflect its ITV.

In the present study, the region of interest in normal lung tissues was outlined, and the SUV of each voxel was computed. The SUV values were normally distributed and recorded as x±SD. If the SUV surpassed x+3×SD, the probability of the voxel belonging to a normal lung tissue would be smaller than 0.003. Therefore, x+3×SD served as the threshold value (ThV) for measuring ITV (9) on two-dimensional (2D)-PET images and showed great potential in three-dimensional (3D)-PET images, as well.

## Materials and Methods

### Phantom

Three hollow plastic models (two heart-shaped models and one ball-shaped model) with internal diameters of 26, 46 and 50 mm and volumes of 18.9, 64.6 and 100.9 mL were used and labeled as model 3, model 2 and model 1, respectively (10). A barrel was filled with 5.9 kBq/mL ^18^F-FDG solution to represent a normal lung tissue. Afterwards, the models were filled with 55.0 kBq/mL ^18^F-FDG solution to simulate a tumor tissue; the models were fixed on the bottom of the barrel (9).

### Simulation of respiratory motions

**Figure 1:**
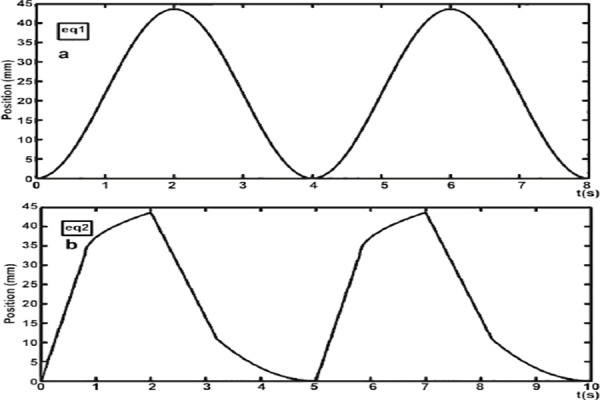
Illustration of time-position curves of the phantom. The phantom movements controlled by stepper motors (following equations 1 and 2a-2d) were graphed in sub-images (based on equations 1 and 2), respectively


To simulate respiratory patterns (i.e., breathing extent and frequency) in the majority of patients by three stepper motors, the phantom was linearly translated, based on the equation (1) or equations (2a–2d), as illustrated in Figure 1. The motors continued moving step by step, and the time interval was set at 0.04 s or 0.05 s for equation (1) or equations (2a-2d), respectively:

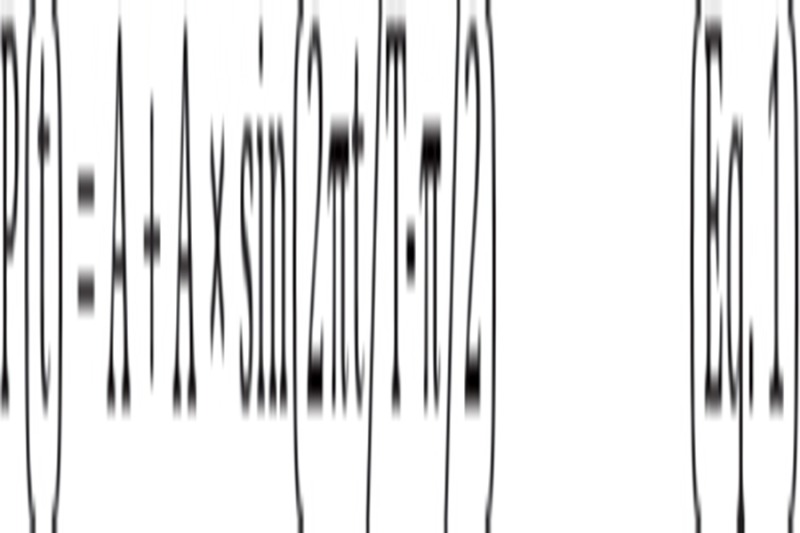


where the amplitudes of motion A were 5.5, 10.9 and 21.8 mm, respectively. The period T for the breathing cycle was set at 4 s.

When 0+5×N-cycle≤ t ≤ 0.4T_i_+5×N-cycle:


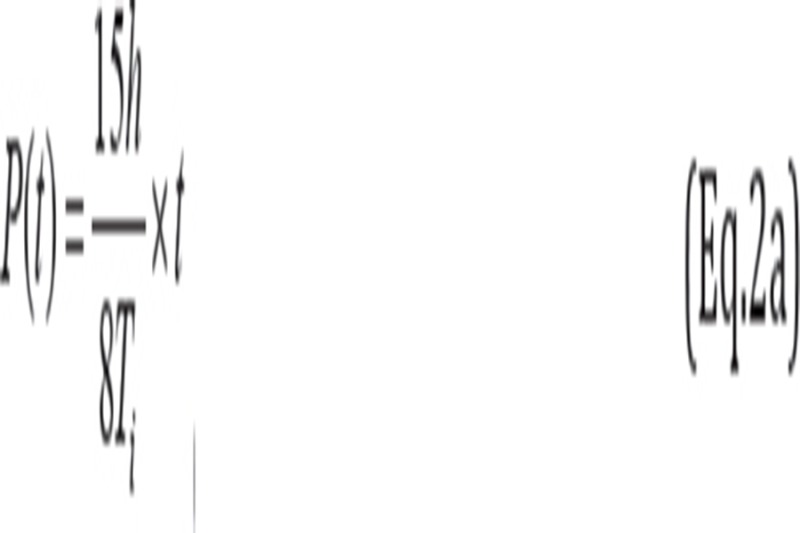


When 0.4T_i_+5×N-cycle<t ≤ T_i_+5×N-cycle:


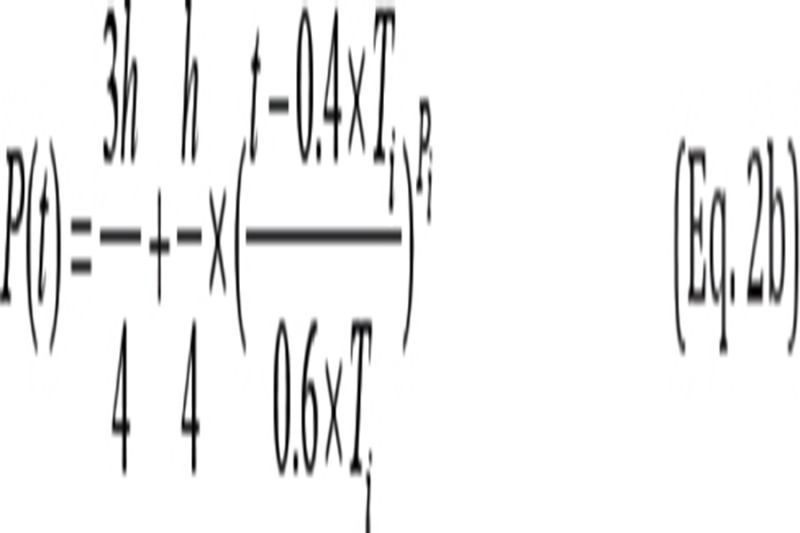


When T_i_ +5×N-cycle< t ≤ T_i_ + 0.4T_e_+5×N-cycle:


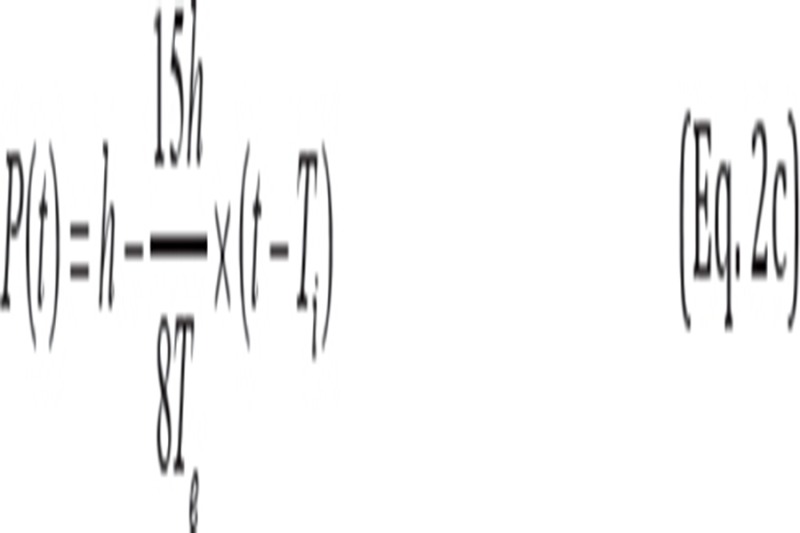


When T_i_ + 0.4T_e_ +5×N-cycle< t ≤ T_i_ + T_e_+5×N-cycle:


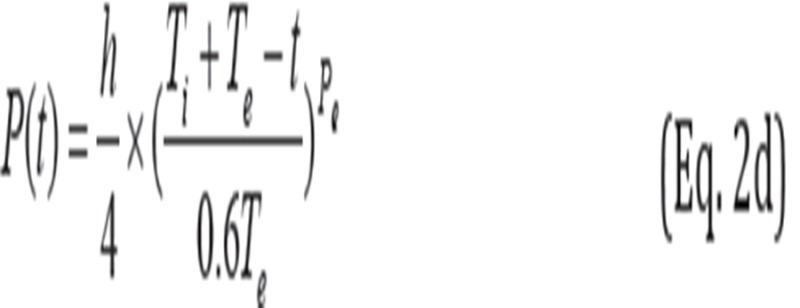


where P(t) is the position of phantom at time t. The maximum motion distance h was 10.9, 21.8 and 43.7 mm, respectively. The inhalation time T_i_ was fixed at 2 s and the exhalation time T_e_ was fixed at 3 s. Therefore, the respiratory cycle was set at 5 s in equations 2a-2d. In addition, P_i_ and P_e_ were 0.5 and 2, respectively. Also, N-cycle denoted “non-negative integer” (e.g., 0, 1 and 2).

### PET imaging and SUV calculation

The image acquisition protocol was in accordance with Chen YCH et al. (11), performed by the PET/CT scanner (Discovery ST, GE Healthcare, USA). The CT scans were obtained with 140 kV, 150 mA, and 0.8 s/rotation, using a 3.75 mm-thick section. Two dimentional (2D) and 3D PET scans were immediately performed following the CT scan with 3.5 min per table position. The Full width at half maximum (FWHM) values were 6.9 and 7.2 mm for 2D and 3D PET acquisitions, respectively.

The PET/CT data of the moving phantom were acquired, following the PET/CT scan at rest. The scan time is presented in Table 1. The CT images were displayed by a 512×512 matrix with a pixel size of 0.98 mm, while the PET images were displayed in a 128×128 matrix with a pixel size of 4.7 mm. The SUVs were calculated, according to Meirelles GS et al. (12).

**Table 1:** Moment of PET data acquisition after the injection

Simulated breath		PET scan	
Motion	Distance (mm)	2D (min)	3D (min)
Equation (1)	10.9	210/223/230	347/353/358
	21.8	192/198/204	331/336/341
	43.7	172/178/185	314/320/325
Equation (2)	10.9	276/282/288	397/403/409
	21.8	257/264/270	381/387/392
	43.7	237/244/250	364/371/376

### ITV of the models on CT images

True GTV (GTV_true_) denotes the volume of the hollow model (11), and ITV was defined as a region, encompassed of GTV_true_ motions during PET/CT data acquisition.

Regarding phantom movements, equations (1) and (2a–2d) were followed, and the exact position of the phantom at a specific moment during movement could be easily located. The CT images at rest could be linearly translated to the determined position, with a weighted factor of 0.01. The overlaying of these weighted CT images resulted in artifact-free CT images of the phantom at motion, covering a whole breathing cycle.

The contours of the ITVs of tumor models could be delineated on the obtained artifact-free CT images, with an optimal Hu threshold value (ThV). The ThV (Hu) matched the volume of the ITV (ITV_true_) of model 2 (ball-shaped), which could be computed using the following equation:


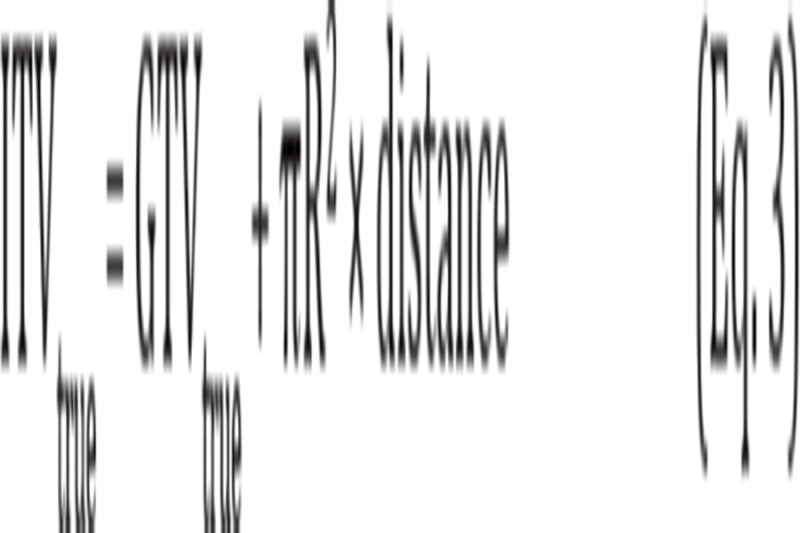


where R can be calculated based on the following equation:


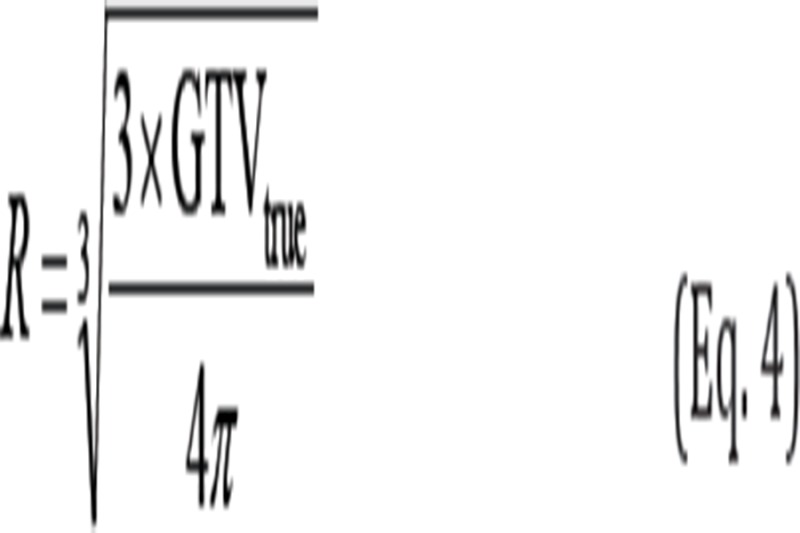


### ITV of the models on PET images

The CT and PET images were accurately co-registered in a single gantry without external markers or internal landmarks (13, 14). After the CT voxels were resized by interpolation to match the PET voxels, the ITV_true_ values of models on PET images were established. Any voxel with an SUV above x+3×SD would be related to the measured ITV (ITV_measured_) of each model.

### Statistical analysis

The volume recovery coefficient (VRC) of the ITV was calculated by the following equation:


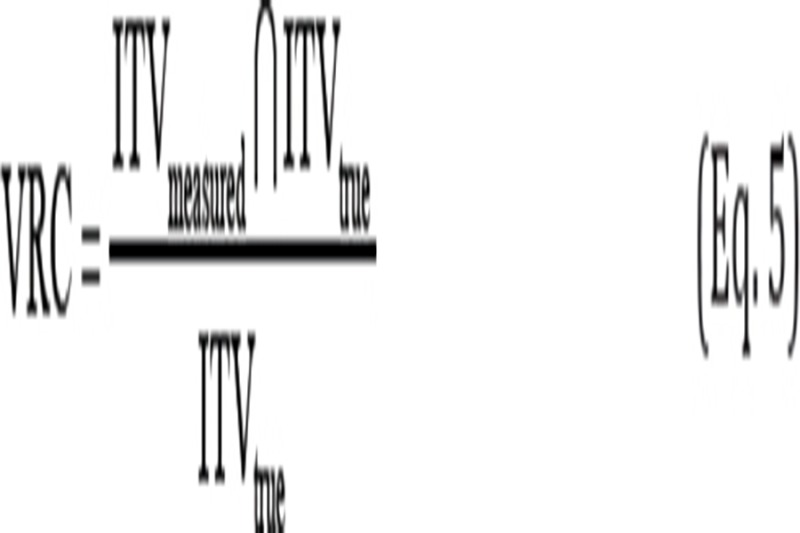


The ITV_measured_ and VRC were calculated for each model and motion by 2D and 3D scan acquisitions. The average values and standard deviations were calculated for further analysis. Nine ITV_true_ values were obtained in this study, and paired t-test was performed to identify significant differences between ITV_true_ and ITV_measured_.

Factorial analysis of variance (ANOVA) was performed to evaluate significant differences between VRCs and factors such as GTV_true_ of the models (models 1, 2, and 3), distance (10.9, 21.8 and 43.7 mm), motion (according to equations 1 and 2) and PET acquisitions (2D and 3D scans). Student-Newman-Keuls test was also performed when the factorial ANOVA reached a significance level of *P*≤0.05 (two-tailed).

## Results

### ITV of the models

The SUV threshold value (x+3×SD) was within the range of 1.7-2.2 for the segmentation of PET images from the models in motion. The ITV_measured_ values as calculated by x+3×SD were not significantly different from ITV_true_ values (*P*>0.05) (Table 2).

**Table 2:** ITV_measured_ (delineated by x+3×SD on PET images) and ITV_true_ of three lung tumor models moving in six different motions.

			ITV_measured_ (mL)			
GTV (mL)	Distance (mm)	ITV_true_ (mL)	Following equation (1)		Following equation (2)	
			2D	3D	2D	3D
100.9	10.9	139.9	156.0 ± 4.8	149.1 ± 2.7	144.3 ± 5.8	144.1 ± 4.8
	21.8	172.7	182.9 ± 5.0	169.1 ± 1.1	170.0 ± 8.4	157.9 ± 3.5
	43.7	234.8	218.1 ± 3.0	210.1 ± 3.8	212.9 ± 3.6	208.3 ± 16.8
64.6	10.9	85.8	108.0 ± 2.6	101.9 ± 1.8	98.4 ± 6.8	100.5 ± 3.8
	21.8	107.0	122.6 ± 3.4	118.3 ± 2.2	120.3 ± 7.5	111.6 ± 2.9
	43.7	149.6	155.8 ± 1.9	152.4 ± 6.4	152.6 ± 9.6	151.3 ± 10.4
18.9	10.9	32.2	39.8 ± 0.8	38.4 ± 1.6	36.0 ± 3.6	37.4 ± 1.6
	21.8	42.8	48.7 ± 2.7	44.8 ± 0.8	44.1 ± 2.3	41.7 ± 2.0
	43.7	63.7	61.2 ± 0.4	56.6 ± 2.3	59.1 ± 2.8	58.7 ± 7.3
			*P* = 0.10	*P* = 0.75	*P* = 0.78	*P* = 0.66

### VRCs of ITVs

**Figure 2:**
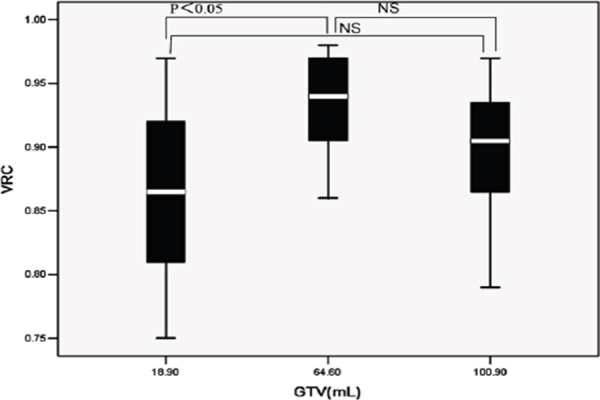
Volume recovery coefficients of three lung tumor models (NS: not significant)

The VRC values are presented in Table 3. These VRCs were significantly different among GTV_true_ values, used for delineating ITVs (*P*<0.01). The VRCs of model 2 surpassed those of model 3 (P<0.05); however, neither model 2 nor model 3 significantly differed from model 1 (*P*>0.05), as indicated in Figure 2.

**Table 3:** Volume recovery coefficients of the ITV_measured_ for the three lung tumor models moving in six different motions.

		Volume recovery coefficient			
GTV (mL)	Distance (mm)	Following equation (1)		Following equation (2)	
		2D	3D	2D	3D
100.9	10.9	0.91 ± 0.01	0.95 ± 0.01	0.94 ± 0.01	0.93 ± 0.01
	21.8	0.97 ± 0.01	0.92 ± 0.01	0.88 ± 0.03	0.87 ± 0.01
	43.7	0.90 ± 0.02	0.86 ± 0.02	0.82 ± 0.02	0.79 ± 0.01
64.6	10.9	0.89 ± 0.01	0.98 ± 0.00	0.97 ± 0.02	0.98 ± 0.01
	21.8	0.97 ± 0.00	0.96 ± 0.01	0.94 ± 0.02	0.93 ± 0.01
	43.7	0.93 ± 0.01	0.92 ± 0.01	0.88 ± 0.02	0.86 ± 0.01
18.9	10.9	0.82 ± 0.01	0.93 ± 0.01	0.91 ± 0.02	0.97 ± 0.00
	21.8	0.94 ± 0.03	0.88 ± 0.01	0.89 ± 0.03	0.85 ± 0.02
	43.7	0.85 ± 0.01	0.81 ± 0.03	0.76 ± 0.01	0.74 ± 0.01

**Figure 3:**
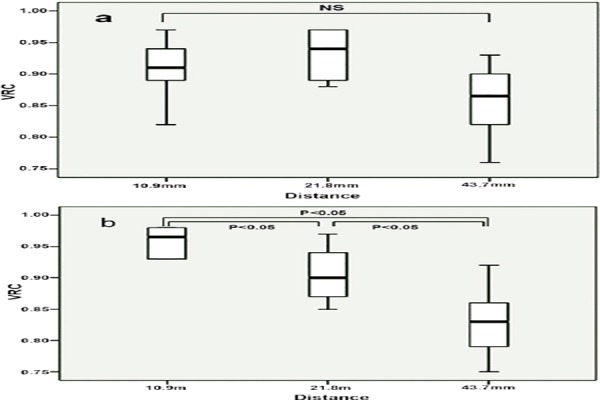
The impact of distance and the acquisition model on volume recovery coefficients of lung tumor models: A) 2D-PET data acquisition, B) 3D-PET data acquisition (NS: not significant)

**Figure 4:**
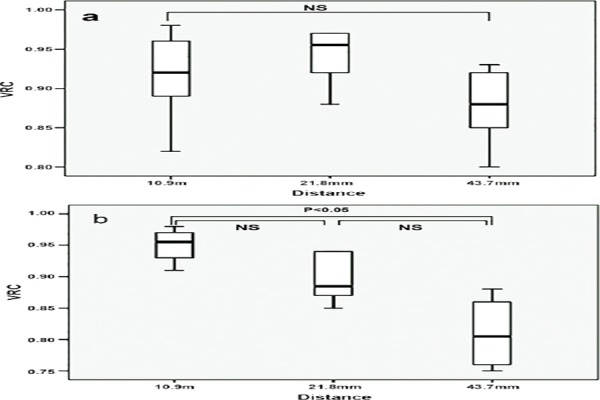
The impact of distance and mode of motion on volume recovery coefficients of lung tumor models: A) Motion of models according to equation 1, B) Motion of models according to equation 2 (NS: not significant)

In addition, statistically significant relationships were found (*P*<0.01) between motion distance, PET acquisition and motions. In the 2D PET scans, the motion distance did not affect VRC (*P*>0.05), as shown in Figure 3A, whereas VRC decreased with increasing motion distance (*P*<0.05) in 3D PET scans, as depicted in Figure 3B. The VRCs decreased with increasing motion distance calculated by equation (2), and the VRCs for 43.7 mm distance were significantly lower than VRCs for 10.9 mm distance (*P*<0.05), as shown in Figure 4. No significant differences were detected between other mean values (*P*>0.05).

## Discussion

Lung tumor and normal tissues are usually surrounded by each another. Therefore, the ThV of SUV in a normal lung tissue was speculated to outline the ITV of the tumor tissue, surrounded by the normal tissue on PET images. We confirmed this hypothesis and showed that x+3×SD could be an ideal threshold for measuring ITV, as ITV_measured_ was not significantly different from ITV_true_.

**Figure 5:**
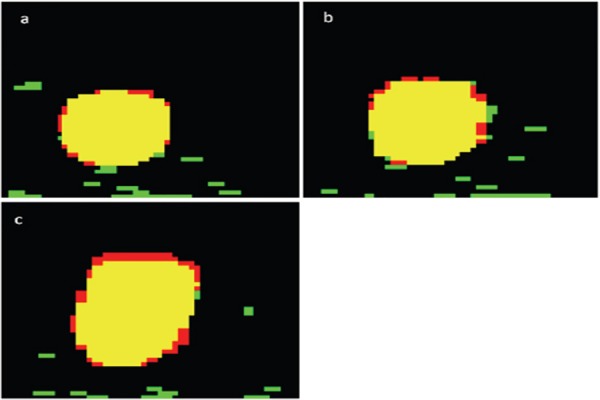
Differences between ITV_true_ and ITV_measured_ obtained by x+3×SD with the maximum distances of A) 10.9 mm, B) 21.8 mm and C) 43.7 mm. ITV_true_ and ITV_measured_, calculated by x+3×SD, and their intersection set are labeled as red, green and yellow, respectively

Furthermore, according to the Gaussian distribution, only 0.1% of the voxels from the normal lung tissue was added to the ITV_true_. However, the ITV_measured_ as determined by x+3×SD calculation did not exactly match the ITV_true_ (Figure 5). Two major reasons can be stated for this discrepancy. First, the SUV of the voxels on the margin of the ITV may be lower than the ThV value. Second, the registered error between CT and PET images, obtained from the same PET-CT scanner, could reach half the size of PET pixels (14).

Since the reference images of ITVs in our study were obtained from simulated CT images and error propagation rules, the registered error between the ITV_measured_ on PET images and ITV_true_ on reference images might be larger than the registered error between CT and PET data acquisitions at rest (i.e., half the size of PET pixels). Therefore, the ITV_measured_ did not match the ITV_true_, and the VRCs were always smaller than one.

The VRCs of model 2 exceeded those of models 1 and 3 (*P*<0.05). One possible reason is that the relative error measurement of the GTV of model 2 (∝1/diameter = 1/50) was smaller than the GTVs of models 1 and 3 (∝1/diameter= 1/46 and 1/26, respectively) for the same CT or PET image. The order of the relative error measurement of ITVs in these three models was similar to the GTV values.

In 2D-PET scans, the motion distance did not affect VRC (*P*>0.05), whereas in 3D-PET scans, VRC decreased by increasing motion distance (*P*<0.05), which was in line with the results reported by Park and colleagues (15).

Two important reasons can be stated in this regard. First, the axially-angled segments yielded truncated views of the object in 3D-PET acquisitions, and these truncated views were smaller in the y direction, whereas 2D transverse planes could be reconstructed independently from each other (16). Second, the registered error and attenuation corrected error between PET and CT images could be enlarged as the motion distance of the models increased. Therefore, since the densities of the models were similar to the liquid in the background, the attenuation corrected error could be neglected.

The VRCs decreased by increasing motion distance calculated by equation (2), and the VRC for 43.7 mm distance was significantly lower than the VRC for 10.9 mm distance (P<0.05). On the other hand, VRCs did not significantly change by increasing motion distance, based on equation (1).

The obtained findings suggest that different motion types may affect the ITV delineation, and four-dimensional PET-CT data acquisition would be beneficial for delineating ITVs as their motion distance increases, since the superior temporal resolution helps to freeze motion images in several phases. 

### Limitations

Considering the partial volume effects, small spheres (<18.9 mL) were not included in this study. In addition, only regular respiratory motions were simulated. Therefore, the effects of patient’s irregular breathing on ITV segmentation in PET images should be further investigated. Moreover, since the normal lung tissue region was manually outlined, the SUV (x±SD) of this region might have been affected by inter- and intra-operator variability.

The heterogeneous tracer distribution in the models was also not simulated in this study, considering the difficulty of simulation in phantom studies. It should be mentioned that the fluid-based background could imitate ^18^F-FDG distribution in normal lung tissues, although it could not mimic its density. Finally, since PET image reconstruction was based on its corresponding CT image (8), the SUV in the realistic lung tumor might have been underestimated, whereas the ITV measured by the background-based method might have been overestimated in this study. 

## Conclusion

The ThV (x+3×SD) of the SUV, determined in the normal lung tissue region, especially on 2D-PET images, had the potential to delineate the ITV of a lung tumor, surrounded by a normal lung tissue for radiotherapy.

## Conflicts of interest

There were no conflicts of interest.
